# miRNAs in NK Cell-Based Immune Responses and Cancer Immunotherapy

**DOI:** 10.3389/fcell.2020.00119

**Published:** 2020-02-25

**Authors:** Silvia Pesce, Marco Greppi, Elisa Ferretti, Valentina Obino, Simona Carlomagno, Mariangela Rutigliani, Fredrik B. Thoren, Simona Sivori, Patrizio Castagnola, Simona Candiani, Emanuela Marcenaro

**Affiliations:** ^1^Department of Experimental Medicine, University of Genoa, Genoa, Italy; ^2^Center of Excellence for Biomedical Research, University of Genoa, Genoa, Italy; ^3^Histological and Anatomical Pathology Unit, Department of Laboratory and Service, E.O. Galliera Hospital, Genova, Italy; ^4^Tumor Immunology Laboratory (TIMM) Laboratory at Sahlgrenska Cancer Center, Department of Infectious Diseases, Institute of Biomedicine, University of Gothenburg, Gothenburg, Sweden; ^5^IRCCS Ospedale Policlinico San Martino, UO Bioterapie, Genoa, Italy; ^6^Department of Earth Science, Environment and Life (DISTAV), University of Genoa, Genoa, Italy

**Keywords:** human NK cells, NK cell receptors, microRNA, immune checkpoint, immunotherapy, gene expression

## Abstract

The incidence of certain forms of tumors has increased progressively in recent years and is expected to continue growing as life expectancy continues to increase. Tumor-infiltrating NK cells may contribute to develop an anti-tumor response. Optimized combinations of different cancer therapies, including NK cell-based approaches for targeting tumor cells, have the potential to open new avenues in cancer immunotherapy. Functional inhibitory receptors on NK cells are needed to prevent their attack on healthy cells. Nevertheless, disruption of inhibitory receptors function on NK cells increases the cytotoxic capacity of NK cells against cancer cells. MicroRNAs (miRNAs) are small non-coding RNA molecules that target mRNA and thus regulate the expression of genes involved in the development, maturation, and effector functions of NK cells. Therapeutic strategies that target the regulatory effects of miRNAs have the potential to improve the efficiency of cancer immunotherapy. Interestingly, emerging evidence points out that some miRNAs can, directly and indirectly, control the surface expression of immune checkpoints on NK cells or that of their ligands on tumor cells. This suggests a possible use of miRNAs in the context of anti-tumor therapy. This review provides the current overview of the connections between miRNAs and regulation of NK cell functions and discusses the potential of these miRNAs as innovative biomarkers/targets for cancer immunotherapy.

## miRNAs as Key Regulators of Gene Expression

About 2,000 human miRNAs are currently recognized. These are small RNAs which originate from longer precursors (Pri-miRNAs) mainly transcribed by the RNA polymerase II (Macfarlane and Murphy, 2010). These Pri-miRNAs undergo a precisely coordinated maturation process involving several steps. In the nucleus, the RNAse III Drosha, supported by the DiGeorge critical region 8 (DGCR8), converts them in short hairpin intermediates of 70–120 nucleotides-long (Pre-miRNAs) (Gregory et al., 2004). After transport to the cytoplasm by exportin 5 (Yi et al., 2003), Pre-miRNAs are then processed by the RNAse III Dycer into mature miRNAs which are duplexes of approximately 22 nucleotides. One strand of the duplex is incorporated along with the argonaute protein in the miRNA-induced silencing complex RISC (Diederichs and Haber, 2007). These complexes promote the pairing of miRNA nucleotide sequences to their target sequences on 3′ UTR sequences of mRNAs and RISC cofactors mediate site-specific cleavage, degradation of the target mRNA, or inhibition of its translation in protein (Gu and Kay, 2010). It is well-known that miRNA can repress the expression of hundreds of different mRNAs. Furthermore, as several different miRNA target sequences may be present on the 3′ UTR of a single mRNA, complex networks of cooperative regulation by several miRNAs may affect the stability or the translation of a multitude of mRNAs (Filipowicz et al., 2008; Liu et al., 2009, 2014). As miRNA recognition sequences appear to be present on most of the protein-coding human mRNAs, the role of miRNAs as regulators of gene expression is quite relevant in mammalian development physiology and pathology (Dallaire and Simard, 2016; Peng and Croce, 2016; Ivanova et al., 2018; Johnson, 2019; Horsburgh et al., 2017). Therefore, miRNAs and in particular those present in body fluids and blood, either as free molecules or included in extracellular vesicles, are receiving increasing attention as possible disease biomarkers (Mori et al., 2019).

## NK Cells as Innate Immune Cells With a Key Role in Fighting Viral Infections and in the Surveillance Against Malignant Transformation

Natural Killer (NK) cells represent cytotoxic, innate lymphoid cells (ILCs) (Cortez and Colonna, 2016), and their main function is to provide the organism with a rapid immune response against infections, autologous transformed cells, and allogeneic cells (Vivier et al., 2011; Del Zotto et al., 2017; Freud et al., 2017). In fact, NK cells do not need to be primed with antigens to become fully functional and the mechanisms of non-self recognition do not rely on genomic recombination and subsequent cell clone expansion events.

Nowadays it is recognized that these cells mediate immune-surveillance also via regulatory functions by secreting cytokines, primarily interferon-γ (IFN-γ) and tumor necrosis factor-α (TNF-α), and by interacting with other immune or adaptive immune cells (Marcenaro et al., 2006; Vivier et al., 2011; Riise et al., 2015; Pesce et al., 2017b; Bernson et al., 2019). In turn, NK cells can respond to different types of chemokines and cytokines produced by other immune cells (Marcenaro et al., 2005a,b, 2006; Moretta et al., 2006; Parodi et al., 2015; Pesce et al., 2016).

NK cells are not a homogeneous population, but there are different NK subsets that differ in phenotype, maturational step, and functions. Among circulating mature NK cells, two main subsets can be identified: regulatory NKs (CD56^bright^/CD16^−^), which are the most abundant in secondary lymphoid organs (SLO) and display the ability to secrete high amounts of pro-inflammatory cytokines, and cytotoxic NKs (CD56^dim^/CD16^+^), which represent about 90% of circulating NK cells (Farag and Caligiuri, 2006; Carrega and Ferlazzo, 2012; Del Zotto et al., 2017).

SLO have been suggested being the anatomical sites where NK cells complete their maturation process that is associated with the transition from a CD56^bright^ to a CD56^dim^ phenotype, acquisition of self-tolerance and lytic activity (Romagnani et al., 2007; Yu et al., 2010).

Both the killing and immune-regulative functions of NK cells depend on a balance of activating or inhibiting signals that originate from NK receptors (NKRs) (activating NKR-aNKR and inhibitory NKR -iNKR-, respectively). The iNKRs include the human leucocyte antigens (HLA) class I-specific Killer Ig-like receptors (KIRs) recognizing allotypic determinants shared by groups of classical HLA-ABC alleles (Moretta et al., 1996), the leukocyte immunoglobulin-like receptor subfamily B member 1 (LILRB1) that is specific for different HLA-class I molecules (Cosman et al., 1997), and the CD94/NKG2A heterodimer specific for HLA-E (Braud et al., 1998) ad well as additional non-HLA-I specific inhibitory receptors, including programmed cell death protein 1 (PD-1), T cell immunoreceptor with Ig and ITIM domains (TIGIT), T-cell immunoglobulin domain and mucin domain 3 (TIM-3), lymphocyte-activation gene 3 (LAG-3), and CD96 (Di Vito et al., 2019). The activating NKRs (aKIRs) include non-HLA-specific receptors such as NCRs (NKp30, NKp44 and NKp46), NKG2D, DNAM-1, NKp80, CD59, NTB-A, and 2B4 (Moretta et al., 2001) as well as the activating HLA class I-specific Killer Ig-like receptors and the HLA-E specific CD94/NKG2C heterodimer. NK cells can also express different Toll-like receptors (TLR), including TLR2, TLR3, TLR5, TLR7, TLR8, and TLR9 (Sivori et al., 2004; Hart et al., 2005; Tsujimoto et al., 2005; Marcenaro et al., 2008; Voo et al., 2014). These receptors, by recognizing conserved pathogen structures, induce NK cell activation (Della Chiesa et al., 2014).

During NK cell development/differentiation, CD94/NKG2A is the first HLA-I specific receptor to be expressed. It appears on the most immature CD56^bright^ CD16^neg/dim^ NK cell subset. After several maturation steps, CD56^bright^ cells become CD56^dim^ CD16^+^, lose NKG2A and acquire KIR and LILRB1 receptors (Di Santo, 2006; Freud and Caligiuri, 2006; Romagnani et al., 2007). The most mature NK cells are KIR^+^ (and/or LILRB1^+^), NKG2A^−^ CD16^bright^ and express the marker of terminal differentiation, CD57 (Moretta et al., 2004; Bjorkstrom et al., 2010; Marcenaro et al., 2017).

Under homeostatic conditions, NK cells continuously receive inhibitory signals mainly originating from the interaction between iNKRs and a large spectrum of classical and non-classical HLA-I molecules expressed on the surface of autologous cells (self-cells). Allogeneic or viral-infected or tumor cells often downregulate or lack altogether the expression of these antigens and therefore fail to be recognized as self-cells by the iNKRs. Under these conditions, the signaling from the aNKRs, engaged with ligands displayed on target cells, prevails, and the NK-mediated killing of these non-self cells is unleashed. Notably, in tumors that maintain the expression of HLA-I molecules, the iNKRs function as immune checkpoints and block the cytotoxic activity of NK cells (Romagne et al., 2009; Vey et al., 2012; Kohrt et al., 2014).

Several strategies have been forwarded to strenghten NK cell activity against HLA-I-expressing cancer cells. For example, IL-2-based immunotherapy allows NK cells to override inhibitory signals from acute myeloid leukemia (AML) blasts (Hallner et al., 2019), and recently, immunotherapies based on the use of therapeutic monoclonal antibodies specific for iNKRs, in particular anti-pan-KIR2D (lirilumab) (Romagne et al., 2009; Kohrt et al., 2014; Vey et al., 2018) and anti-NKG2A (monalizumab), have been developed (André et al., 2018; Tinker et al., 2019; Zaghi et al., 2019). These agents efficiently disrupt the interaction between these NK cell immune checkpoints and their ligands, and will in this way enable NK cells to efficiently kill also HLA-I^+^ tumor cells (Chiossone et al., 2017; Di Vito et al., 2019).

In addition, the microenvironment of chronic infections and tumors may lead to NK cell phenotypic changes and impairment of NK cell functions (Bi and Tian, 2017). The most frequently NK cell phenotypic changes are represented by downregulation of the aNKRs expression (Costello et al., 2002; Romero et al., 2006; Carlsten et al., 2009; Pesce et al., 2015; Han et al., 2018; Poznanski and Ashkar, 2019) and/or upregulation/*de novo* expression of iNKRs (Carlsten et al., 2009; Di Vito et al., 2019; Sanchez-Correa et al., 2019). In fact, it has been unveiled that besides T lymphocytes also NK cells can express PD-1, an immune checkpoint specific for the PD-L1/2 molecules often displayed on the surface of tumor cells (Pesce et al., 2019b).

PD-1 is expressed on a subset of fully mature (KIR^+^CD57^+^NKG2A^−^) NK cells from one-fourth of human cytomegalovirus (HCMV) seropositive individuals (Della Chiesa et al., 2016; Pesce et al., 2017a; Mariotti et al., 2019). Increased proportions of PD-1^+^ NK cells can be observed in patients affected by different types of tumors (Beldi-Ferchiou et al., 2016; Pesce et al., 2017a, 2019a,b; André et al., 2018). Accordingly, studies suggest a role for NK cells in immunotherapy targeting the PD-1/PD-L1 axis (Hsu et al., 2018) and this is clinically relevant for patients with tumors characterized by a T cell resistant (HLA-I^neg^) phenotype.

Apart from the wide-spread use of checkpoint inhibitors in melanoma, lung cancer etc., agents blocking the PD-1/PD-L1 axis are currently being evaluated in clinical trials on both hematologic and solid tumors as monotherapy or in combination with other agents, including other forms of immune checkpoint blockade, such as anti-panKIR2D and anti-NKG2A antibodies in the case of HLA-I^+^ tumor cells (Moretta et al., 1996, 2001; Cosman et al., 1997; Braud et al., 1998; Sivori et al., 2004; Marcenaro et al., 2008; Di Vito et al., 2019).

In summary, NK cell activation depends on the nature of interactions between inhibitory/activating receptors on their surface and the relative ligands on target cells, and thus receptor/ligand pairs could represent key checkpoints in the regulation of anti-tumor NK cell activity and in the planning of innovative NK cell-based immunotherapy.

## miRNAs as Regulators of NK Cells Survival, Development/Maturation, and Functions

Numerous studies showed that miRNAs play a relevant role in the regulation of NK cell survival, development/maturation, activation, proliferation, cytotoxicity, and cytokine production both in healthy and pathological conditions (i.e., tumors/viral infections) by targeting receptors or factors involved in transcriptional expression (Table 1).

**Table 1:** Examples of miRNAs expressed in NK cells and involved in the modulation of several aspects of NK cell development and functions.

	**miRNAs**	**Induced by**	**Inhibited by**	**Targets**	**Effects**	**References**
**miRNAs involved in NK cell differentiation/development**	miR-150^¶^			Myb	Promotes the development of NK cells	Bezman et al., 2011
	miR-181a/b			NLK	Promotes the development of NK cells ↑ INF-γ production	Cichocki et al., 2011
	miR-583			IL2Rγ	↓ NK cell differentiation	Yun et al., 2014
**miRNAs involved in the regulation of NK cell functions**	miR-27a-5p		IL-15	GzmB Prf1	↓ NK killing activity	Kim et al., 2011
	miR-30e		IFN-α	Prf1	↓ NK killing activity	Wang et al., 2012
	miR-378		IFN-α	GzmB	↓ NK killing activity	Wang et al., 2012
	miR-150		IL-15	Prf1	↓ Prf1 ↓ NK killing activity	Kim et al., 2014
	miR-362-5p^¬^			CYLD (neg. reg. of NF-kb)	↑ Expression of: IFN-gamma, perforin, granzyme-B, and CD107a	Ni et al., 2015
	miR-155^‡^	IL-2, IL15 or IL-21			↑ NK killing activity	Liu et al., 2012
	miR-155	IL-12, IL-15, IL-18		SHIP-1	↑ NK killing activity ↑ INF-γ production	Sullivan et al., 2013
	miR-99b miR-330-3p^$^				NK cell activation but diminished cytotoxicity	Petty et al., 2016
	miR-1245	TGFß		NKG2D	↓ NK killing activity	Espinoza et al., 2012
	miR-183	TGFß		DAP12	Destabilization of 2DS4 and NKp44 ↓ NK killing activity	Donatelli et al., 2014
	miR-218-5p		IL-2	SHMT1	↓ IFN-γ and TNF-α production ↓ Cytotoxicity	Yang et al., 2019
**Pathogens-modulated miRNAs in NK cells**	miR-15a^†^		EBV-encoded latent membrane protein (LMP1)	Myb Cyclin D1	Growth arrest	Komabayashi et al., 2014
	miR-155	IL-12 and IL-18 via STAT4		Noxa (early post MCMV); SOCS1 (late post MCMV)	↑ Antiviral immunity	Zawislak et al., 2013
	miR-29a-5p	HCV		PU.1Prf1	↑ miR-155 ↓ Prf1 ↓ NK killing activity	Elemam et al., 2015
**miRNAs in tumor-associated NK cells**	miR-183^∧^	TGFß		DAP12	Destabilization of 2DS4 and NKp44 ↓ NK killing activity	Donatelli et al., 2014
	miR-1245	TGFß		NKG2D	↓ NK killing activity	Espinoza et al., 2012
	miR-218-5p		IL-2	SHMT1	↓ IFN-γ and TNF-α production ↓ Cytotoxicity	Yang et al., 2019
	miR-150			DKC1AKT2	↑ Apoptosis in tumor cells ↑ Tumor suppression	Watanabe et al., 2011
	miR-203		Promoter methylation in lymphoma		Tumor suppression	Chim et al., 2011
	miR-494-3p			PTEN	AKT activation	(Chen et al., 2015)
	miR-142-3p^§^			RICTOR	Suppression of AKT	(Chen et al., 2015)
	miR-155			SHIP1	↓ Cell survival and Cell-cycle progression	Yamanaka et al., 2009
	miR-21			PTEN; PDCD4	↓ Cell survival (anti-apoptotic)	Yamanaka et al., 2009
	miR-26a/b miR-28-5 miR-30b miR-101 miR-363		c-Myc	MUM1, BLIMP1, and STMN1 in NKTL	↓ Cell growth (NK/T-cell Lymphoma)	Ng et al., 2011
	miR26a/b			BCL2	↓ Cell growth	Ng et al., 2011
	miR-363 miR-28-5				↓ Cell growth	Ng et al., 2011
	miR-101			STMN1IGF1BCL2	↓ Cell growth	Ng et al., 2011
	miRNA-10a miRNA-342-3p			TIAM1	Low miRNA expression correlated with development of Extranodal NK/T-cell lymphoma	Huang et al., 2016
	miR-221				Poor Survival in Plasma NK/T-cell Lymphoma	Guo et al., 2010
	miR-155			BRG1	Activation of STAT3/VEGFC signaling and promotion of NKTCL viability and lymphangiogenesis	Chang et al., 2019
**miRNAs involved in the regulation of NK cell immune checkpoints**	miR-182^#^			NKG2D? NKG2A?	↑ Cytotoxicity via Prf1 counter intuitive effects on NKG2D and NKG2A	Abdelrahman et al., 2016; El Sobky et al., 2016
	miR-146a-5p^°^			KIR2DL1 KIR2DL2	↑ NK killing activity	Pesce et al., 2018
	miR-26b-5p miR-26a-5p miR-185-5p			KIR3DL3	NK cell activation?	Nutalai et al., 2019

¶ *Controls iNKT cells development and apoptosis (Bezman et al., 2011; Winter and Krueger, 2019) and has negative effects on acute T-cell lymphoblastic leukemia (T-ALL) survival (Saki et al., 2015) whereas it has a protective effect on CD4^+^ and CD8^+^ T cells by controlling the expression of pro-apoptotic genes (Cron et al., 2019)*.

¬ *Promotes malignancy of chronic lymphocytic leukemia (CLL) (Yang et al., 2015)*.

‡ *Reported to be also involved in CD8+ T cell activation (Gracias et al., 2013) and T cell development*.

$ *Also involved in the inhibition of TGF-β expression in CD8+ Treg cells (Rouas et al., 2019)*. *Also involved in the control of chronic lymphocytic leukemia clonal expansion (Cutrona et al., 2017)*.

∧ *See also involvement of miR-183C (Ichiyama et al., 2016) and miR-183-5p in Th17 cytokine production and Th17/Treg imbalance in thrombocytopenia (Hua et al., 2019), respectively*.

§ *Also involved in CD25+ CD4 T cell proliferation by targeting the expression of GARP (Zhou et al., 2013)*.

# *Also Promotes clonal expansion of activated T helper lymphocytes (Stittrich et al., 2010)*.

° *Promotes growth of acute leukemia cells (Wang L. et al., 2019)*.

### miRNAs Involved in NK Cell Differentiation/Development

The first evidence of the important role played by miRNAs within the immune system was provided by genetic studies showing a critical requirement for Dicer *in vivo*. Conditional deletion of Dicer in various hematopoietic lineages in mice produced defects, such as impaired cell differentiation, proliferation, and survival (Muljo et al., 2005; Cobb et al., 2006; Koralov et al., 2008; Liston et al., 2008; Fedeli et al., 2009).

Bezman et al. (2010) investigated the role of miRNAs by ablation of the miRNA biogenesis pathway, through deletion of Dicer or Dgcr8 in the mature murine peripheral NK cells. Dicer- and Dgcr8- deficient NK cells showed an increased cell death supporting the important role of miRNAs in controlling cell survival. Moreover, Dicer- and Dgcr8-deficient NK cells are able to respond efficiently through their cytokine receptors; however, function of their immunoreceptor tyrosine-based activation motif (ITAM)-containing aKIRs is impaired.

By using another molecular approach, Sullivan et al. (2012) eliminated Dicer during the earliest stages of murine NK cell development in the bone marrow to better characterize the phenotypic features derived from global loss of mature miRNA expression. These studies confirmed that the absence of miRNAs led to reduced numbers and percentages of NK cells, and a decreased *in vitro* survival/proliferation.

Studies utilizing next-generation sequencing in mouse and human reported some information about the miRNA repertoire in resting CD56^+^ CD3^−^ human or NK1.1^+^CD3^−^ murine NK cells and upon cytokine activation (Fehniger et al., 2010; Liu et al., 2012; Wang et al., 2012). Furthermore, Ni et al. (2015) identified the miRNA profiles of human NK cells from different compartments (peripheral blood, cord blood, and uterine decidua). Very recently, our group, by analyzing peripheral blood NK cells from 10 different human healthy donors, identified a 108 miRNA signature able to discriminate CD56^bright^ from CD56^dim^ NK cell subsets independently from their surface phenotype (Pesce et al., 2018). Interestingly, we found some miRNAs (miR-146a-5p, miR-92a-3p, miR-223-3p, miR-873-5p, miR-31a-5p, hsa-miR-130a-5p, miR-181a-2-3p) with consistent differential expression in the two NK cell subsets, and with an intermediate expression in the CD56^bright^/CD16^dim^ NK cell subset, which represents a transition phase in the NK cell maturation process of NK cells.

A key miRNA for NK cell development is miR-150. A gain of function miR-150 transgene in mouse was demonstrated to drive the development and maturation of NK cells. In line with this, mice with a targeted deletion of miR-150 instead display cell lineage–intrinsic defect in their ability to generate mature NK cells (Bezman et al., 2011).

Additional miRNAs relevant for NK cell development and maturation are miR-181 and miR-583. Cichocki and coworkers found that miR-181 promotes NK cell development via inhibition of the Nemo like kinase (NLK) (Cichocki et al., 2011) while Yun and collaborators showed that the miR-583 targets and downregulates IL2Rγ in NK cells acting as a negative regulator of their differentiation process (Yun et al., 2014).

### miRNAs Involved in the Regulation of NK Cell Functions

Accumulating evidence suggests that distinct miRNAs may play regulative roles on NK cell functions both in terms of cytotoxicity and cytokine production. In this context, miR-27a-5p (Kim et al., 2011), miR-378, miR-30e (Wang et al., 2012), and miR-150 (Kim et al., 2014), were proposed as negative regulators of NK cell killing ability. In particular, miR-378 was found to target granzyme b (Gzmb) (Wang et al., 2012), miR-30e and miR-150 have as target perforin (Prf1) (Wang et al., 2012; Kim et al., 2014) while miR-27a-5p targets both (Kim et al., 2011). By contrast, Prf1, Gzmb, IFN-γ, and CD107a in human NK cells were all upregulated after miR-362-5p overexpression. Ni and collaborators indeed found that this miRNA targets the mRNA coding for the cylindromatosis lysine 63 deubiquitase (CYLD) and suggested that miR-362-5p promotes NK cell effector functions (Ni et al., 2015).

miR-155 should also be included among the miRNAs that enhance NK cell functions. In particular, IL-2, IL-15, and IL-21 upregulate this miRNA, which in turn, enhances NK cell cytotoxicity (Liu et al., 2012). Moreover, miR-155 extensively regulates the NK cell activation threshold regulating molecules involved in NK cell activation and their IFN-γ release by modulating the expression of the phosphatase SHIP-1, T-bet/Tim-3, or the activation of several signaling pathways, including those involving PI3K, NF-kB, and calcineurin (Trotta et al., 2012; Sullivan et al., 2013; Cheng et al., 2015). Similarly, miR-181 was also found to promote IFN-γ production in primary NK cells in response to cytokine stimulation through regulation of the Notch pathway (Cichocki et al., 2011).

In a study aimed to identify miRNAs potentially involved in the pathogenesis of chronic fatigue syndrome or myalgia encephalomyelitis in peripheral blood mononucleate cells (PBMC), 34 miRNAs were found upregulated compared to healthy controls and 2 of these (miR-99b, miR-330-3p) were confirmed having the most important deregulation in NK cells in terms of cytotoxic activity (Petty et al., 2016).

### Pathogens-Modulated miRNAs in NK Cells

The host's immune responses must be strictly regulated by an sophisticated balance between positive and negative signals during the fight against pathogens.

One of the mechanisms by which pathogens can break this balance is that of interfering with the regulatory role of miRNAs.

TLRs are receptors of the innate immune system that directly recognize conserved structures of both viral and bacterial origin that are present and functional on NK cells (Della Chiesa et al., 2014). It has been recently demonstrated that several miRNAs, including miR-21, miR-146, miR-155, and let-7 family can bind to TLRs (acting also as physiological ligands for these receptors) or proteins in TLR signaling pathways. These interactions can regulate the expression and the transcriptional responses of TLRs (Bayraktar et al., 2019). In addition, some miRNAs and miRNA-containing exosomes can selectively activate innate immune effector cells, including NK cells, via the TLR1–NF-kB signaling pathway (He et al., 2013).

Enhancement of NK cell cytotoxicity with upregulation of Prf1 was described as associated with miR-182 overexpression in NK cells derived from hepatocellular carcinoma (HCC) patients (Abdelrahman et al., 2016). However, a subsequent study from the same group reported contradicting roles of this miRNA in both NK cells and in hepatocytes infected by hepatitis C virus (HCV) (El Sobky et al., 2016).

Komabayashi and collaborators demonstrated that the Epstein-Barr virus (EBV)-encoded latent membrane protein 1 (LMP1) is able to downregulate the expression of the miR-15a and increase MYB and cyclin D1 in cell lines with an NK cell phenotype (Komabayashi et al., 2014), thus suggesting that miR-15a may have a role in the repression of NK cell proliferation. In line with this, Cheng and collaborators found that the down-regulation of miR-155 suppressed IFN-γ production through Tim-3 signaling and lead to HCV evading immune clearance (Cheng et al., 2015). However, the specific target of miR-155 in the context of these two studies remains unknown.

miR-155 was found to be induced by IL-2 and IL-18 via STAT4 and able to reduce the expression of NOXA and SOCS1 at distinct stages of homeostasis and activation. As NK cells of mice with a targeted miR-155 deletion displayed dramatically diminished effector activities and reduce memory cell numbers in both lymphoid and non-lymphoid tissues afterwards murine cytomegalovirus (MCMV) infection, these findings suggest that miR-155 promotes antiviral immunity (Zawislak et al., 2013). However, a study by Elemam et al. (2015) reached contrasting conclusions as they observed that HCV infection might abolish NK cell cytotoxicity via modification of PU.1 (a key transcription factor in the NK cell development), and Prf1/NKG2D expression by miR-29a-5p and miR-155 overexpression, respectively.

### miRNAs in Tumor-Associated NK Cells

Several studies demonstrated that miRNAs might also act as oncogenes or tumor suppressor genes in different human cancer histotypes. Most of the endogenous miRNAs that have been characterized so far modulate NK cell antitumor activity in the tumor microenvironment (TME). TGFß released by tumor cells in the TME is a powerful inhibitor of the NK cell killing activity, and Donatelli and collaborators have shown that a specific miRNA, miR183, is induced by TGFß (Donatelli et al., 2014). They also formally proved that this miRNA downregulates the expression of the DNAX activating protein 12 kDa (DAP12) that was found critically involved in the stabilization of KIR2DS4 and NKp44 receptors on the plasma membrane and required for their signaling activities. Interestingly, loss of DAP12 was also identified as a common trait in tumor-infiltrating lymphocytes in lung cancer (Donatelli et al., 2014). TGFß has also been reported to increase post-transcriptionally the levels of mature miR-1245 which suppresses NKG2D expression, thus spoiling NKG2D-mediated immune responses and enhancing the tumor supporting properties of the TME (Espinoza et al., 2012).

Yang and collaborators reported that miR-218-5p suppresses the NK-mediated killing of lung adenocarcinoma by targeting Serine Hydroxymethyltransferase 1 (SHMT1) (Yang et al., 2019).

Several studies identified miRNAs (miR-203, miR-494-3p, miR-142-3p, miR-155, miR-21) that affect NK cell lymphoma survival and apoptosis modulating different pathways including the PTEN-AKT-mTOR pathway (Yamanaka et al., 2009; Chim et al., 2010, 2011; Ichimura et al., 2010; Chen et al., 2015).

Recent findings provided evidence on the role of some miRNAs as tumor suppressors, such as miR-150 that is involved in the pathogenesis of malignant lymphoma, by increasing the incidence of apoptosis and reducing cancer cell proliferation (Watanabe et al., 2011).

Several studies were performed using as model the NK/T cell lymphoma (NKTL), a progressive malignancy with unfavorable prognosis without a specific treatment, and most of them were pursued to identify dysregulated miRNAs that can affect targets involved in the oncogenesis of NKTL. In this context, Ng and collaborators found that miR-26a, miR-26b, miR-28-5, miR-30b, miR-101, and miR-363 were downregulated, possibly via MYC, in NKTL and NK cell lines compared to normal NK cells and that the suppressed miRNA expression allowed increased expression of genes implicated in oncogenesis (Ng et al., 2011). Furthermore, in a recent study focused on NKTL, Huang and collaborators reported data suggesting that miR-10a and miR-342-3p may be implicated in the development of NKTL through the T-lymphoma invasion and metastasis inducing factor 1 (TIAM1) pathway, which has a crucial role in the development of several types of human cancer (Huang et al., 2016). Other miRNAs such as miR-221 and miR-155, associated with promotion of NKTL viability, have been proposed as potential molecular markers of NKTL (Guo et al., 2010; Chang et al., 2019).

Recently, growing evidence has shown that extracellular vesicles (EVs) released by NK cells transport miRNAs capable of exerting a strong anti-tumor effect in immunosuppressive TME (Fabbri, 2020). Previous studies have also demonstrated that NK cell-derived exosomes have tumor-specific accumulation with no cytotoxic activity against normal tissues (Lugini et al., 2012). Meanwhile, microenvironment acidic pH promotes the traffic of this EVs in tumor cells (Parolini et al., 2009). In addition, NK cell-derived exosomes also exhibit the benefits of being stable vesicles and maintain their biological activities. Thus, NK cell-derived exosomes can both facilitate tumor targeting and act as direct antitumor agent. These properties make them more suitable for clinical applications, thus suggesting a possible use of NK-cell derived EVs as anticancer agents as a new avenue for tumor therapy (Wang H. et al., 2019).

### miRNAs Involved in the Regulation of NK Cell Immune Checkpoints

Immune checkpoints have a key role in regulating the intensity of immune responses of lymphocytes by performing inhibitory functions. The use of immune checkpoint inhibitors in immunotherapy has driven anti-cancer treatment on a novel level. Emerging evidence suggests that some miRNAs can control the expression of immune checkpoints on the surface of NK cells or that of their ligands on tumor cells. This suggests a possible use of miRNAs in the context of anti-tumor therapy.

**Figure 1:**
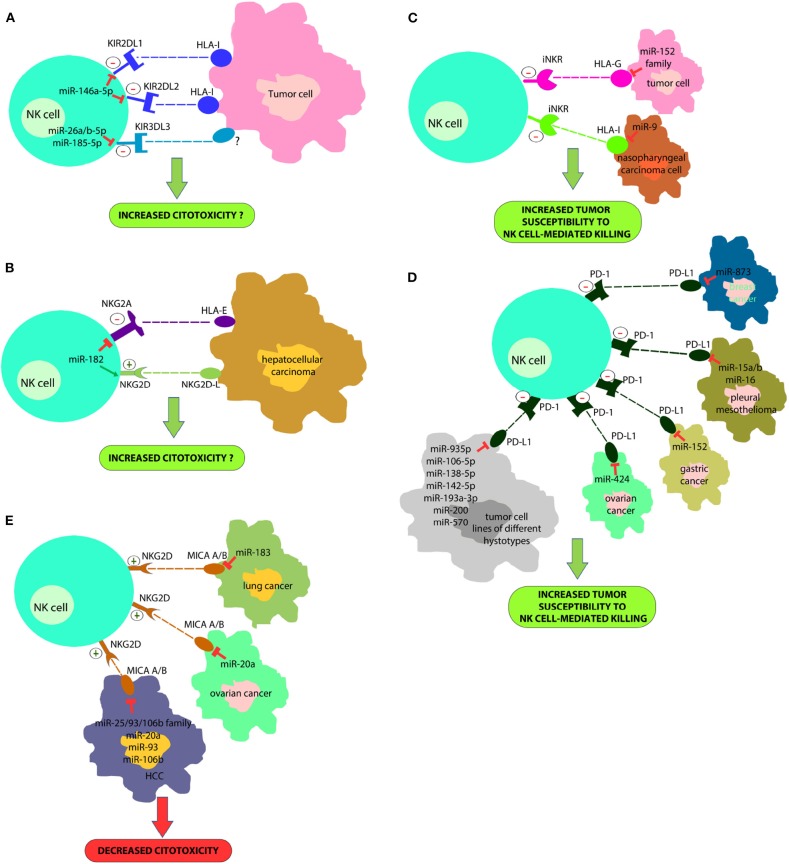
A new strategy for improving NK cell-based immunotherapy: miRNAs can directly regulate the expression of different NK cell immune checkpoints (including KIRs, PD-1, NKG2A, and other iNKR) **(A,B)** or their ligands (HLA-I, PD-L1, HLA-E/G) **(C,D)**. In addition, some miRNAs can also regulate the expression of activating NK cell receptors (i.e., NKG2D) or their ligands (e.g., MIC A/B) **(E)**. This effect can deeply impact on NK cell ability to recognize and kill cancer cells. In particular, a downregulation of immune-checkpoints or immune-checkpoints' ligands can restore an appropriate NK cell anti-tumor activity; on the contrary, a downregulation of activating receptor expression or their ligands can affect their anti-tumor potential. This suggests innovative miRNA-based therapeutic approaches to unleash NK cell effector functions in the cancer treatment.

In a recent study we proved that the miR-146a-5p is able to downregulate both KIR2DL1 and KIR2DL2, two HLA-specific inhibitory receptors belonging to KIR family (Pesce et al., 2018) (Figure 1A). Furthermore, *in silico* functional characterization of miR-146a-5p gene targets, identified CD94, HLA-C, HLA-E, Prf1, and several other KIRs genes as additional targets. These results are in line with the higher levels of miR-146a-5p found in CD56^bright^ NK cells and with other studies suggesting that this miRNA is engaged in the regulation of NK cell maturation via the STAT1 (Xu et al., 2017) and NF-kappaB (Wang et al., 2018) signaling pathways. A different research group identified very recently three miRNAs, miR-26a-5p, miR-26b-5p, and miR-185-5p, as inhibitors of the expression of an additional KIR, the KIR3DL3, which is included in the iNKRs group but it is still poorly characterized (Nutalai et al., 2019) (Figure 1A). Therefore, the role of these miRNAs in NK cells development or function remains to be defined.

The study of Abdelrahman and collaborators showed that enhancement of NK cell cytotoxicity by miR-182 in human hepatocellular carcinoma and increased Prf1 expression were indirect effects likely mediated by a complex modulation of NKG2D and NKG2A levels in these cells at different stages of the disease (Abdelrahman et al., 2016) (Figure 1B).

Regarding miRNAs regulating PD-1 expression, miR-28 (Li et al., 2016), miR138 (Wei et al., 2016), and miR-4717 (Zhang et al., 2015) have been found to target this immune checkpoint, and to induce T cell exhaustion. It has been demonstrated that miR-4717 play a role in chronic Hepatitis B Virus (HBV) infection, where this miRNA is significantly reduced (Zhang et al., 2015). Since also NK cells may express PD-1, it cannot be excluded that these miRNAs may play an important regulatory role also in these innate cells.

Notably, some mi-RNAs target additional immune checkpoints playing a critical role for cytotoxic immune cell functions, such as miR-28 targeting TIM-3 (Li et al., 2016), expressed by both T and NK cells (Di Vito et al., 2019), and miR-16, miR-138, and miR-195 targeting cytotoxic T-lymphocyte antigen 4 (CTLA-4), mainly expressed by T cells (Wei et al., 2016; Tao et al., 2018).

### miRNAs Involved in the Regulation of the Expression of Ligands for NK Cell Receptors

Tumor immune evasion is not restricted to the upregulation of immune checkpoint proteins, but also to the dysregulation in the expression of immune checkpoint ligands, including classical and non-classical HLA-I molecules or ligands for activating NK cell receptors. In this context, it has been found that miR-9 is involved in the downmodulation of the expression of HLA-I molecules in human cancer cells, preventing the detection of cancer cells by the immune system (Gao et al., 2013) (Figure 1C). This suggests that tumors overexpressing this miRNA might become resistant to CD8^+^ T-cell mediated killing but susceptible to NK cell-mediated attack. In addition, the tumor-suppressive miR-148 family has been found to regulate the expression of HLA-G, a ligand for different NK cell inhibitory receptors (Mandelboim et al., 1997; Seliger, 2016) (Figure 1D).

Recently, it has been demonstrated that some miRNAs directly target the 3′-UTR of PD-L1 mRNA and others the PD-1/PD-L1 indirectly by targeting the related signaling pathways (Wang et al., 2017; Gao et al., 2019; Omar et al., 2019). miR-15a, miR-15b and miR-16 were discovered to downregulate the PD-L1 expression in malignant pleural mesothelioma cell line (Kao et al., 2017) (Figure 1D). miR-34a was found to be inversely correlated with PD-L1 expression in 44 AML samples (Wang et al., 2015) (Figure 1D). miR-935p, miR-106b-5p, miR-138-5p, miR-142-5p, miR-193a-3p, miR-200, and miR-570 overexpression downregulate PD-L1 in tumor cell lines of different hystotypes (Chen et al., 2014; Guo et al., 2015; Cioffi et al., 2017; Jia et al., 2017; Kao et al., 2017) (Figure 1D). miR-152 was found to regulate PD-L1 in gastric cancer tissues (Guo et al., 2015), while miR-424 regulates the PD-L1 expression in chemo-resistant ovarian cancer patients (Xu et al., 2016) (Figure 1D). Notably, miR-873 decreased the stemness and resistance to chemotherapy of breast cancer cells, depending on PD-L1 and the downstream PI3K/Akt and ERK1/2 signaling, by directly inhibiting PD-L1 expression. This suggests that miR-873/PD-L1 regulatory axis may represent a new therapeutic target in breast cancer. Such data are interesting for employing miRNAs as useful diagnostic targets and valuable biomarkers for prognosis in the PD-1/PD-L1 blockade therapy. miR-155, a key component of inflammatory responses, is dysregulated in different cancer cell types. In this context, it has been recently demonstrated that the induction of miRNA-155 expression, suppresses the expression of PD-L1 in both primary lymphatic endothelial cells and fibroblasts, by exposing these cells to the TNF-a and IFN-γ proinflammatory cytokines (Yee et al., 2017).

Moreover, different miRNAs downregulate MHC class I chain-related protein A/B (MICA/B) expression (NKG2D NK-cell receptor ligands) and this represents another mechanism of immune suppression targeting NK cells cytotoxicity. These miRNAs include miR-183 that targets MIC A/B in lung cancer (Trinh et al., 2019), miR-20a that induces the same effect in ovarian cancer (Xie et al., 2014) and miR-25/93/106b family, miR-20a, miR-93 and miR-106b that act in HCC (Kishikawa et al., 2013) (Figure 1E).

## Concluding Remarks

Recently, there has been a substantial evolution in cancer therapy, mainly oriented toward immunotherapy approaches, in substitution or in combination with classical therapy. Cancer immunotherapy represents a promising new era in cancer management due to the relatively high safety margins and selectivity, compared to the classical cancer chemotherapeutic agents.

miRNAs have come to light over the last years as key actors in epigenetic regulation and for their capacity to modulate tumor immunity by directly regulating the expression of genes involved in the activation or suppression of the immune response. In this review, we focused our attention on the current state of knowledge concerning the involvement of miRNAs in various physiologic processes of NK cells. In particular, we discussed their abilty to regulate NK immune responses and their potential implications in resistance to cancer immunotherapy, with main focus on immune checkpoints. In this context, several miRNAs have been found to modulate different immune checkpoints/ligands interaction, including the PD-1/PD-L1 axis or their upstream genes. Future studies comparing miRNAs' expression profiles in patients who respond to immune checkpoint blockade immunotherapies as compared to non-responders will help to disclose the potential role of miRNAs as non-invasive predictive biomarkers for monitoring the response and clinical outcomes to immunotherapy with immune checkpoint inhibitors.

## Author Contributions

All authors listed have made a substantial, direct and intellectual contribution to the work, and approved it for publication.

### Conflict of Interest

The authors declare that the research was conducted in the absence of any commercial or financial relationships that could be construed as a potential conflict of interest.
